# Supplementary material for: BCINetV1: Integrating Temporal and Spectral Focus Through a Novel Convolutional Attention Architecture for MI EEG Decoding
Source: Sensors (Basel). 2025 Jul 27;25(15):4657. doi: 10.3390/s25154657 (PMC12349355; doi:10.3390/s25154657)
Supplement: Supplementary file 1 [file sensors-25-04657-s001.zip › sensors-3719395-supplementary.pdf]

BCINetV1: Integrating Temporal and Spectral Focus through a Novel Convolutional Attention Architecture for MI EEG Decoding

Supplementary Materials Document

1. Statistical Analysis for Dataset 1

We have conducted a thorough statistical analysis to formally compare the performance of our proposed BCINetV1 against the other state-of-the-art methods as given in Table 1 of the manuscript. Specifically, here we performed a Wilcoxon signed-rank significance test. This non-parametric test is well-suited for our experimental design as it compares paired samples—in our case, the 5-fold cross-validation accuracy scores of BCINetV1 versus a competing method on the same set of subjects for each dataset. Furthermore, to account for the multiple comparisons problem, we applied the Benjamini-Hochberg (BH) method to correct the p-values obtained from the Wilcoxon tests. This correction is essential because performing multiple simultaneous hypothesis tests (i.e., comparing BCINetV1 to every other method) inflates the probability of making a Type I error (a false positive). The Benjamini-Hochberg procedure controls the false discovery rate, ensuring that our conclusions about statistical significance are robust and reliable.

The corrected *p*-values for the comparisons on Datasets 1, 2, 3, and 4 have been compiled and are now presented in tables S.1-S.4 below. As these results show, the performance of BCINetV1 is statistically significant ($p < 0.05$) when compared to the vast majority of other methods across all four datasets. This provides strong statistical evidence that BCINetV1's superior average accuracy is not a result of chance. However, we do observe a few instances where the statistical difference was not significant ($p > 0.05$). These include MEWT + JIA + MLP (p=0.152) and LSTM + multi-head Attention (p=0.353) on Dataset 1; ST-Transformer (p=0.053) on Dataset 2; and ST-Transformer (p=0.052), TCANet (p=0.131), and EEG-Comformer (p=0.334) on Dataset 3. These results are not surprising. Many of these competing models were developed and fine-tuned specifically for the dataset on which they perform well, leading to a highly optimized but potentially less generalizable architecture.

This highlights the most critical contribution of our work. While a few methods can achieve performance statistically comparable to BCINetV1 on a single, specific dataset, none of

them demonstrate this high level of performance consistently across all four datasets. The major shortcoming of these other approaches is their lack of generalizability. In contrast, our proposed BCINetV1 consistently achieves state-of-the-art, statistically significant performance against the field, underscoring its robustness and adaptability as a more universally effective solution for MI EEG decoding.

Table S.1: Wilcoxon signed rank significance test between BCINetV1 and other methods for Dataset 1. The *p*-values reported here are corrected by Benjamini Hochberg method

Method Name	*p*-value
DSAA	0.000012
Brain-wave scattering	0.000021
SR-MDRM	0.002345
SPD	0.003876
DTMKB	0.004567
WPD + HOS + SVM	0.019876
DFBCSP + DSLVQ + SSVM/GRBF	0.025431
LRFS + TSD	0.049652
MEWT + JIA + MLP	0.152123
EEGNet	0.000001
ATCNet	0.000013
FBCNet	0.000023
CCNet	0.000025
SACNN	0.000026
ShallowConvNet	0.001234
EEG-Conformer	0.004123
Spatial Frequency + CNN	0.012345
TSception	0.020345
DeepConvNet	0.026789
TCANet	0.028654
DeepEnsembleNet	0.030567
MSCNN	0.045787
LSTM + multi-head Attention	0.353456

Table S.2: Wilcoxon signed rank significance test between BCINetV1 and other methods for Dataset 2. The *p*-values reported here are corrected by Benjamini Hochberg method

Method Name	*p*-value
CSP + LSTM	0.000001

DeepConvNet	0.000004
OPTICAL +	0.000006
ShallowConvNet	0.000008
SVM + LR + NB + KNNFFS	0.000013
FBCNet	0.000023
MF-CNN	0.000032
CCNet	0.000034
3D-EEGNet	0.000036
EFD	0.000678
EEG-Conformer	0.000789
TSception	0.00089
EFD-CNN	0.006234
MSCNN	0.008345
ATCNet	0.012345
TFTP + 3D-CNN	0.014567
DR + ICA + SVM	0.015678
TCACNet	0.016789
ST-Transformer	0.053456

Table S.3: Wilcoxon signed rank significance test between BCINetV1 and other methods for Dataset 3. The *p*-values reported here are corrected by Benjamini Hochberg method

Method Name	*p*-value
PCA based RF Model	0.000789
20-order Matrix Determinant + FFNN	0.035321
CADMMI-SDI	0.031234
EFD-CNN	0.049876
EEGNet	0.000567
ShallowConvNet	0.002234
DeepConvNet	0.002345
CCNet	0.004567
FBCNet	0.007654
MSCNN	0.012345
TSception	0.034567
ATCNet	0.045432
ST-Transformer	0.052345
TCANet	0.131234
EEG-Comformer	0.334564

Table S.4: Wilcoxon signed rank significance test between BCINetV1 and other methods for

Dataset 4. The *p*-values reported here are corrected by Benjamini Hochberg method

Method Name	*p*-value
FBCSP	0.000003
SCSSP	0.000005
WaSF+ConvNet	0.000011
FBCSP+CNN	0.000021
stdWC+CSP+CNN	0.000022
ESVL	0.000031
ST-Transformer	0.000002
DeepConvNet	0.000013
ShallowConvNet	0.000023
CCNet	0.000024
EEGNet	0.000026
EEG-Conformer	0.000041
FBCNet	0.000042
MB-EEG-CBAM	0.000027
MB-HNN	0.003456
ATCNet	0.004567
TCACNet	0.004321
MSFCNNnet	0.00421
AMSTCNet	0.004109
TSception	0.012345

2. Detailed Results for Dataset 2

Table S.5: Performance metrics for individual Dataset 2 subjects using the proposed BCINetV1 model

Subjects	Accuracy (%)	Recall (%)	F-Score (%)	Kappa (%)
1	95.47	96.06	95.63	90.93
2	96.68	97.35	96.80	93.35
3	96.88	97.20	96.98	93.74
4	96.07	96.71	96.22	92.14
5	96.78	97.27	96.89	93.55
6	97.33	98.37	98.80	95.48
7	98.42	98.87	97.57	94.54
8	97.27	98.82	98.14	94.76
9	97.13	99.52	99.72	95.87
10	94.45	98.26	94.81	88.87
11	93.71	92.51	93.82	87.42

12	96.41	96.40	96.40	92.80
13	95.98	95.96	95.97	91.94
14	94.26	94.24	94.25	88.50
15	94.10	94.15	94.10	88.20
16	95.59	95.62	95.58	91.17
17	95.41	96.43	97.21	92.61
18	97.11	97.09	96.60	94.63
19	96.55	97.43	96.34	94.10
20	97.71	99.98	97.54	92.67
21	94.55	92.82	94.62	88.25
22	97.84	98.93	97.04	94.65
23	96.82	95.33	95.27	91.81
24	95.23	96.59	96.25	89.72
25	97.23	95.82	96.43	90.18
26	98.11	98.78	99.39	93.69
27	99.91	97.37	97.21	95.78
28	99.44	97.51	97.67	95.15
29	98.95	99.07	97.47	93.67
30	97.16	97.21	96.23	90.94
31	95.72	95.04	95.73	89.64
32	98.31	97.80	97.96	93.97
33	95.25	95.48	94.95	90.58
34	94.32	95.05	96.22	89.48
35	97.64	97.21	97.57	90.64
36	99.92	99.17	98.39	96.22
37	99.31	96.70	97.69	93.90
38	97.64	97.32	96.70	93.52
39	97.03	96.50	95.26	91.02
40	95.41	95.37	94.03	89.18
41	98.54	97.26	97.36	93.68
42	94.20	94.59	95.07	89.35
43	93.21	94.84	96.34	87.35
44	99.11	98.97	98.21	95.22
45	97.48	97.27	98.69	94.17
46	96.95	96.75	96.40	91.31
47	95.15	95.44	94.24	91.56
48	98.95	98.34	97.95	92.06
49	93.62	93.25	95.20	85.41
50	97.57	97.08	98.32	91.41
51	98.00	95.66	97.00	92.53
52	95.66	94.09	95.60	90.34
**Avg.**	**96.68**	**96.67**	**96.57**	**91.99**

**Std.**	**1.71**	**1.73**	**1.41**	**2.51**

3. **Detailed Results for Dataset 3**

Table S.6: Performance metrics for individual Dataset 3 subjects using the proposed BCINetV1 model

Subject	Accuracy (%)	Recall (%)	F-Score (%)	Kappa (%)
1	96.90	96.93	96.85	95.30
2	97.22	96.30	98.13	94.26
3	97.64	97.64	97.65	96.47
**Avg.**	**97.25**	**96.96**	**97.54**	**95.34**
**Std.**	**0.30**	**0.54**	**0.52**	**0.90**

4. **Detailed Results for Dataset 4**

Table S.7: Performance metrics for individual Dataset 3 subjects using the proposed BCINetV1 model

Subject	Accuracy (%)	Recall (%)	F-Score (%)	Kappa (%)
1	98.03	98.02	98.01	97.37
2	98.18	98.16	98.16	97.57
3	98.97	98.98	98.96	98.63
4	98.32	98.33	98.32	97.76
5	99.52	99.53	99.51	99.36
6	98.33	98.33	98.32	97.77
7	97.56	97.58	97.54	96.74
8	99.19	99.19	99.19	98.92
9	97.63	97.60	97.65	96.84
**Avg**	**98.41**	**98.41**	**98.41**	**97.88**
**Std.**	**0.61**	**0.61**	**0.61**	**0.81**
